# A Convenient and Low-Cost Model of Depression Screening and Early Warning Based on Voice Data Using for Public Mental Health

**DOI:** 10.3390/ijerph18126441

**Published:** 2021-06-14

**Authors:** Xin Chen, Zhigeng Pan

**Affiliations:** 1 School of Medicine, Hangzhou Normal University, Hangzhou 311121, China; chenxinboss@stu.hznu.edu.cn; 2 Engineering Research Center of Mobile Health Management System, Ministry of Education, Hangzhou Normal University, Hangzhou 311121, China; 3 Institute of VR and Intelligent System, Hangzhou Normal University, Hangzhou 311121, China

**Keywords:** public health, depression, voice data, decision tree, screening model, early warning

## Abstract

Depression is a common mental health disease, which has great harm to public health. At present, the diagnosis of depression mainly depends on the interviews between doctors and patients, which is subjective, slow and expensive. Voice data are a kind of data that are easy to obtain and have the advantage of low cost. It has been proved that it can be used in the diagnosis of depression. The voice data used for modeling in this study adopted the authoritative public data set, which had passed the ethical review. The features of voice data were extracted by Python programming, and the voice features were stored in the format of CSV files. Through data processing, a big database, containing 1479 voice feature samples, was generated for modeling. Then, the decision tree screening model of depression was established by 10-fold cross validation and algorithm selection. The experiment achieved 83.4% prediction accuracy on voice data set. According to the prediction results of the model, the patients can be given early warning and intervention in time, so as to realize the health management of personal depression.

## 1. Introduction

Depression is a common mental health disease [1,2]. Depressive patients are usually accompanied by depressed mood, loss of interest, mental retardation, voice movement reduction, self-criticism negation, insomnia, lack of appetite, suicidal thoughts, and other symptoms, and their life becomes boring and meaningless [3,4]. Depression has the characteristics of a slow onset, long treatment cycle, high incidence rate, and being difficult to diagnose. The treatment of depression mainly includes psychotherapy, drug therapy, and psychotherapy and drug therapy at the same time. Although the treatment of depression is relatively mature, there are many deficiencies in the diagnosis of depression.

First, the incidence rate of depression is high, but patients are often ashamed to admit that they are suffering from mental illness, and rarely seek to diagnose and treat themselves actively. Secondly, there is subjective deviation in the diagnosis of depression in clinic [5]. At present, the common method of the diagnosis of depression is mainly based on the Diagnostic and Statistical Manual of Mental Disorders (DSM-5), which is subjective by doctors’ interviews with patients and evaluation scales [6,7]. Thus, it is difficult to identify depression, and there are some missed diagnoses and misdiagnoses. Thirdly, there are a lack of low-cost and large-scale screening tools for depression [8], and patients lack the relevant knowledge for the self-assessment of depression. Based on the above reasons, many patients with depression do not know their illness, thus losing the best opportunity for treatment. Therefore, it is an important direction to find an objective method of rapid screening and early warning for depression.

In September 2020, the National Health Commission of China published the work plan of exploring the characteristic services for the prevention and treatment of depression on its official website. The program requires that for high school and college students, depression screening will be included in the students’ physical examination, and will pay attention to the students with abnormal screening results. Moreover, for other groups, such as the elderly, occupational groups, women and children [9], they are also faced with a variety of life pressures. Among the above groups, the people with anxiety and even depression symptoms are also a large group. Therefore, it is an urgent problem to find a fast, convenient, and low-cost screening and early warning method for depression.

As the kind of data that are easy to obtain, voice data have the characteristics of convenience, low cost and objectivity. In recent research literature, some scholars have used voice data to evaluate and diagnose depression [10,11,12]. Research showed that there are obvious differences between the voice characteristics of patients with depression and normal people [13,14]. The spectral- and energy-related features of voice were different, and could be used to classify and predict depression [13]. These two types of characteristics changed with the different degree of depression of the speaker. Voice features could be used to measure depression and mania in bipolar disorder, and could be used as an objective state marker. Moreover, voice features, such as pitch, variance and so on, were used in the study [14]. Therefore, voice data are an ideal data for depression screening, and have unique advantages. In addition to the above advantages, voice data acquisition also has the advantages of non-invasiveness, as patients do not feel any discomfort and do not need to pay any efforts [15].

Antosik-Wojcinska et al. [16] found that the voice data generated in the process of mobile phone calls and mobile phone use could reflect the social and physical activities of the actor, and had the characteristics of objectivity, which could be used as an effective marker to reflect the mood state. Mood is an important diagnostic feature in the diagnosis of depression, so voice data can be used as one of the diagnostic data of depression. Arevian et al. [10] collected voice samples of patients through a voice response system. By calculating the characteristics of speech samples and using machine learning model, they could judge the clinical status of patients with mental illness, including the judgment of depression. Low et al. [2] made a systematic review of the papers on the use of voice data in the evaluation of mental diseases in recent years. It was found that the samples of patients’ voice fragments, voice processing technology, and machine learning prediction model could be used to evaluate depression, schizophrenia and other mental diseases. The methods of data acquisition, feature extraction and machine learning model construction were discussed. Existing studies have shown that the use of voice data for depression screening is a feasible method [17,18].

However, how should voice data be used for depression screening? As well as how to extract voice features and which voice features to extract, how can the accuracy of screening be improved? These problems need further study. There are many kinds of voice data. What kind of voice data are more effective also needs further research. In addition, the existing studies only focus on methods, and the health management of depression needs to take further measures, such as early warning according to the idea of preventive treatment of disease in traditional Chinese medicine.

In this work, we have made the following key contributions:

(1) A whole process of depression screening and early warning method, based on voice data, was designed and implemented;
(2) The features of voice data were extracted by the Python programming language and the feature database was automatically generated;
(3) Early warnings were given to individuals screened as in a depressive state, in order to make them know their health status as soon as possible;
(4) The proposed method has the advantages of convenience and low cost, which can provide a feasible solution for the automatic screening of depression with high accuracy.

## 2. Materials and Methods

### 2.1. Acquisition of Voice Data

The speech data used for depression screening were tested with authoritative depression speech public data set. At present, one of the most famous and newest depression public data sets is the multi-modal open dataset for mental disorder analysis (MODMA) depression public data set released by Lanzhou University in 2020 [19].

In order to ensure the reliability of the data of patients with depression, the selection criteria of patients with depression were as follows: they were diagnosed and recommended by at least one clinical psychiatrist, the score of the patient health questionnaire (PHQ-9) was greater than or equal to the score of 5 [20], and the diagnosed criteria of the mini-international neuropsychiatric interview (MINI) reached the criteria for depression.

This study used the voice database of the MODMA depression public data set. All data were obtained with the informed consent of the participants and the public data set was approved by the related ethics committee. By submitting the application form, the right to use the MODMA data set for depression research was obtained.

The MODMA voice database included the voice data of 52 subjects, including 23 patients with depression and 29 healthy controls. In the patients within the depression group, the age ranged from 18 to 52 years old. Further, in the healthy controls group, the age ranged from 19 to 52 years old. There were 36 males in the voice database, including 16 patients with depression and 20 healthy controls. There were 16 females, of whom 7 were depression patients and 9 healthy controls. Each person had 29 voice data samples, including interview, passage reading, vocabulary reading and picture description. The sampling frequency was 44.1 KHz, the sampling depth was 24 bits, and the data storage format was WAV.

This data set is the scientific research achievement of China 973 program and other large programs, which has good authority and reliability. Because the assessment of depression needs to use the scale, the commonly used scale of depression generally has many questions in order to fully understand the level of depression. Eighteen interviews represent 18 scale questions. Each question is a recording, so the first 18 recording files are relatively short. The detailed description about the 29 voice recordings can be found in the study [19], which can answer what the individual speech samples/tasks represent and why they are defined like that. Voice in this paper refers to the recording voice file and the format is WAV. 

### 2.2. Voice Data Preprocessing

**Figure 1:**
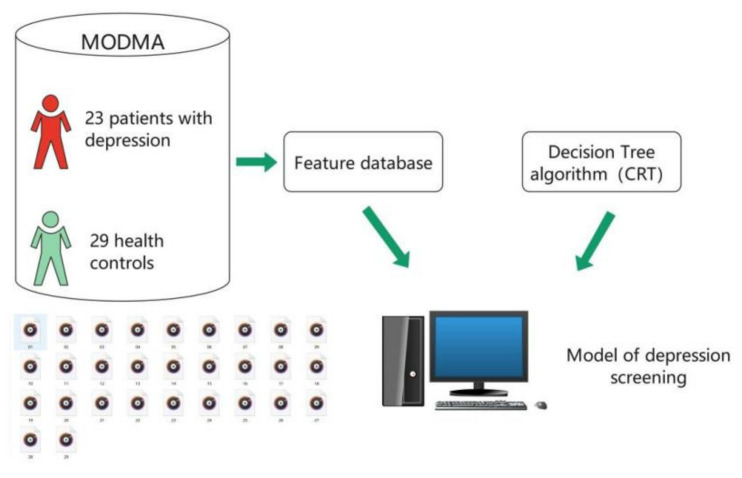
Brief introduction of the generation process of depression screening model.

The principle of machine learning to identify depression is to memorize and calculate the comprehensive characteristics of depressive patients and non-depressive patients through machine learning program, and generate a model. The brief principle is shown in Figure 1. The 29 health controls in Figure 1 refer to the sample of “non-depressive” speakers. The CRT in Figure 1 refers to the classification and regression tree.

In order to recognize voice data and build machine learning model to screen depression, it is necessary to extract the features of speech data. When extracting voice features, the voice data must be preprocessed. Voice data are a kind of data that change with time. According to the length of time, voice data can be divided into frame and utterance. The time of a frame of voice is usually between 10 and 30 ms. Due to the inertia of voice, the signal of a frame of voice is usually considered to be stable and does not change with time. Utterance is a voice sample, which is composed of multiple voice frames. Therefore, each voice sample of the tester is an utterance.

For frame and utterance, their voice features are called low-level descriptors (LLD) [21] and high-level statistics functions (HSF), respectively. LLD feature is the calculation feature for one frame of voice, while HSF feature is the statistical feature obtained by doing statistical operations on the multi-frame voice features of utterance, such as average value, maximum value, etc. Therefore, in order to get the relevant features of the speaker’s voice data, it is necessary to preprocess the voice data by dividing them into frames, and then extract the statistical features as the features of the voice data. Before voice framing, the pre-emphasis and windowing operation should be carried out. The purpose of pre-emphasis is to increase the high-frequency resolution of voice. Preprocessing can be implemented in Python or MATLAB programming language.

### 2.3. Voice Features and Their Extraction Methods

At present, there are the following several famous feature sets used in the field of voice emotion recognition: GeMAPS (the Geneva minimalistic acoustic parameter set) feature set, eGeMAPS feature set [21], Interspeech 2009 challenge feature set, etc.

EGeMAPS feature set is an extension of GeMAPS feature set, which has 88 voice features. The feature set is suitable for acoustic research and emotion computing. It mainly includes the statistical data of pitch, jitter, loudness, mel-frequency cepstrum coefficient (MFCC), harmonics-to-noise ratio (HNR), shimmer, formant and other related characteristics. The features of the feature set have the following characteristics: it can mark the potential emotional physiological changes in the voice data, it has been proved to be valuable in previous studies, and it can be extracted.

The voice features used in this study adopted the eGeMAPS feature set and 88 dimensional features of the voice were extracted for machine learning modeling. Feature extraction was realized by Python programming and calling the openSMILE open-source toolbox. The 88 dimensional voice features were extracted from all the voice files by using cyclic programming.

For machine learning, we only need to calculate the values of these features, then the machine learning model can be trained to predict depression. The calculation of features has open formulas and functions. The detailed descriptions about eGeMAPS features can be found in the study [21]. For machine learning, the characteristics of depressed speech are the comprehensive expression of a series of digital features.

### 2.4. Grouping of Voice Features and Generation of Database

The voice database in the MODMA described in this paper has 52 folders, each named after the participant’s number [19]. There are 29 voice samples in each folder. The files are named 1–29 in a specific order. The details of each voice are shown in Table 1.

Both passage reading and vocabulary reading belong to the reading type data. TAT is also the voice data in the form of picture description, which is classified as picture description data. By adding the for-loop function in the Python program and traversing all the files in the folder, the voice features of 29 voice samples in each folder can be extracted and stored in the CSV file. Using Python program to traverse all 52 folders, we can get a total of 1508 feature files of the voice samples.

In the process of the experiment, the voice data in the folder No. 02010004 were defective. In order to improve the accuracy and reliability of the modeling results, the data group was discarded. Therefore, the feature data of 1479 voice samples in 51 folders were obtained. At the same time, according to the different types of voice files, the feature data were classified and four databases were generated, named all, interview, reading and picture.

The all database contained 1479 voice feature files. The interview database contained 918 voice feature files of all interview types. The reading database contained 357 voice feature files of passage reading and vocabulary reading types. Picture database included voice feature files of picture description and TAT types, containing a total of 204 voice feature files.

### 2.5. Decision Tree Machine Learning Algorithm

Decision tree is a kind of machine learning, which is a prediction model [16,22,23]. The process of decision analysis of decision tree [24] is like the trunk of a tree, which reflects the process of grouping learning data, so it is called decision tree. There are the following three types of nodes in decision tree: root node, leaf node and middle node.

There is only one root node, which includes all training data. Each internal node corresponds to a segmentation condition. The classification results calculated by decision tree are displayed in leaf nodes, which are data sets with classification labels. There are the following three kinds of decision trees: classification tree, regression tree and classification regression tree. Classification tree is used to predict classification-dependent variables, regression tree is used to predict numerical-dependent variables, classification regression tree can establish both classification tree and regression tree. The type of decision tree used in this study was classification tree.

For decision tree model, 10-fold cross validation was used for training data. The extracted feature data were randomly divided into 10 parts, of which 9 parts were used for training, and 1 part was reserved as test data. The above-process was repeated 10 times, using different test data each time. Then the results of the 10 times were summarized, and the average value was taken as the estimation of algorithm performance index. A 10-fold cross validation is a popular algorithm choice at present.

In this study, the decision tree model used the following two algorithms: Chi-squared automatic interaction detector (CHAID), and classification and regression tree (CRT) [25]. CHAID algorithm determines branch variables and segmentation threshold by statistical significance test, and makes decision by generating multiple trees. CRT algorithm determines the branch variables and segmentation threshold based on variance and Gini coefficient, and makes decision by generating binary tree. In this study, the results of CRT algorithm and CHAID algorithm were different in different types of voice database. Therefore, for different types of voice data, the algorithm of decision tree model selection was different, and the algorithm with good results was selected to build decision tree model. Compared with other machine learning algorithms, decision tree is intuitive and easy to understand. For decision tree machine learning in this study, SPSS decision tree tool is used to model automatically.

## 3. Results

### 3.1. The Visualization of Voice Features

**Figure 2:**
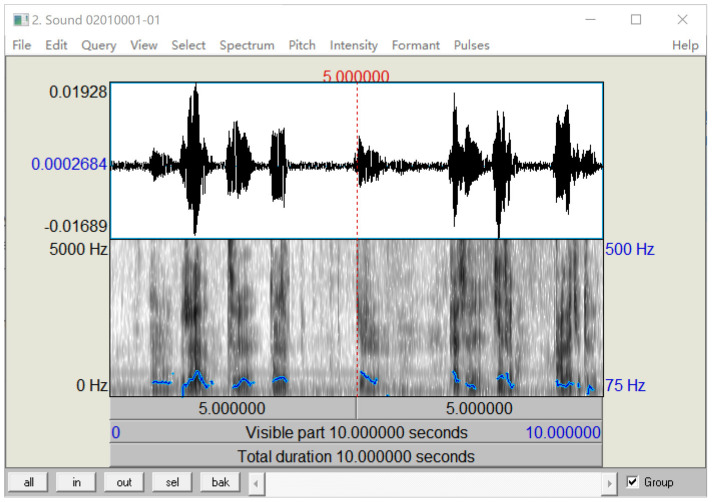
Visualization of voice features.

Praat is a famous software for speech processing. Because the features used in machine learning are digital features, it is not easy to be intuitively understood. Therefore, Praat software is used to visualize the features of a voice sample, which is convenient for preliminary observation. The real calculation of digital features needs a computer algorithm to realize, because the features are various and complex. Some features were visualized, as shown in Figure 2, including the waveform of sound, spectrum and pitch in the software menu. The waveform was easy to observe. The image below the waveform showed spectrum, and the blue curve on the image showed pitch. Here, the Praat diagram shows that voice features can be extracted and visualized.

### 3.2. Scatter Plot of Voice Feature Distribution

For machine learning, 88 dimensional voice features are extracted from the voice data. In addition, two-dimensional features are added to form a 90-column feature database. One is data label, which is represented by V1 in the feature database, indicating whether the data are the voice of a patient with depression, and the value of 1 represents yes and the value of 0 represents no. The other is the location and name information of the voice data file, which is represented by V2 to facilitate data searching.V3-V90 represent the 88 dimensional voice feature values calculated by the Python program. 

**Figure 3:**
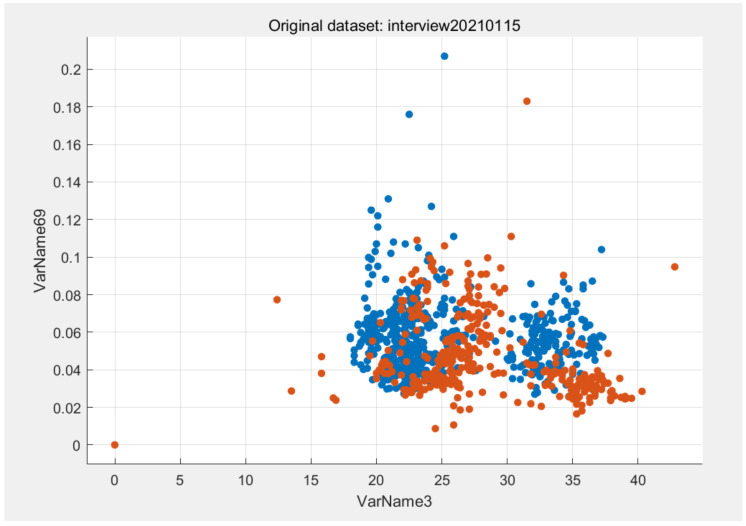
Scatter diagram of V3 and V69 in the interview database. The blue dot means there is no depression. The orange dots represents depression.

V3 and V69 were two of the most important features after calculating the importance of features in the interview database decision tree model. V3 was the first dimensional voice feature in the database, and V69 was the 67th dimensional voice feature. The feature scatter diagram of V3 and v69 is shown in Figure 3. It can be seen that the distribution of features had obvious rules, which was easy to distinguish by algorithm. The abscissa VarName3 represents V3. The vertical coordinate VarName69 represents V69. 

The scatter diagram of V3 and v69 was generated by MATLAB software to observe the distribution of features.

### 3.3. Evaluation Criteria and Classification Results of the Model

#### 3.3.1. Evaluation Criteria of the Model

The classification results of the decision tree model adopt the following indexes: accuracy, precision, recall, specificity, and F1 score [3]. The above-indicators are calculated by a confusion matrix.

The confusion matrix consists of the following four important definitions: true positive (TP), false positive (FP), false negative (FN), and true negative (TN). The evaluation indicators of the confusion matrix are shown in Table 2.

#### 3.3.2. Classification Results of Different Databases

The decision tree model was used to test the following four databases: interview, reading, picture and all. On the interview database, the decision tree model was constructed by using 10-fold cross validation and the CHAID algorithm. Multiple trees were generated for prediction, and the accuracy of the model was 81.8%. If the algorithm of building a decision tree model was changed to the CRT algorithm, to generate a binary tree for prediction, the recognition accuracy of the model reached 83.4%, and the accuracy was improved by 1.6%. Therefore, it is better to use the CRT algorithm to build a decision tree model for the interview-type voice data recognition.

On the reading database, 10-fold cross validation and CHAID algorithm were used to build the decision tree model. The accuracy of the model was 75.4%. By changing the decision tree algorithm to the CRT algorithm, the accuracy of model recognition reached 76.8%, which was improved by 1.4%. Therefore, for the reading-type voice data recognition, using the CRT algorithm to build a decision tree model is better. Using the same method, in the picture database, using the CHAID algorithm to build a decision tree model, the model recognition accuracy was 75%, while using the CRT algorithm, the model recognition accuracy was 73%, reduced by 2%. Therefore, for the picture-type voice data recognition, it is better to use the CHAID algorithm to build a decision tree model.

Finally, the decision tree model was constructed by using 10-fold cross validation on the all database, composed of all types of voice data. When the CHAID algorithm was used, the model recognition accuracy reached 82.4%. When using the CRT algorithm, the model recognition accuracy reached 83.4%. Therefore, for depression recognition of all kinds of voice data, the decision tree model based on the CRT algorithm is better.

The evaluation index results of building a decision tree model on different databases are shown in Table 3, and the value of the result is the model with better accuracy in the CHAID and CRT algorithm.

It can be seen from the data in Table 3 that the performance of the decision tree model constructed by the interview database is close to that constructed by the all database, and is better than that of the reading database and picture database. The complexity of data collection using the all database is much higher than that of the interview database, so the interview-type voice data are an ideal form for depression screening modeling.

### 3.4. Screening and Early Warning of Depression

After the decision tree model of depression was constructed, it could be used for depression screening of the new testers. The model will automatically judge the depression state of the new tester by inputting the specified type of voice data into the model. The predicted value of depressive state is one. If the predicted value is zero, it means that the tester has no depression. For the individuals whose depressive state is identified as one, depression warning will be carried out. Common warning methods include telephone, SMS platform, WeChat platform, mobile app [26], etc. Moreover, depression intervention measures can be started simultaneously [27,28].

### 3.5. The Model of this Study

**Figure 4:**
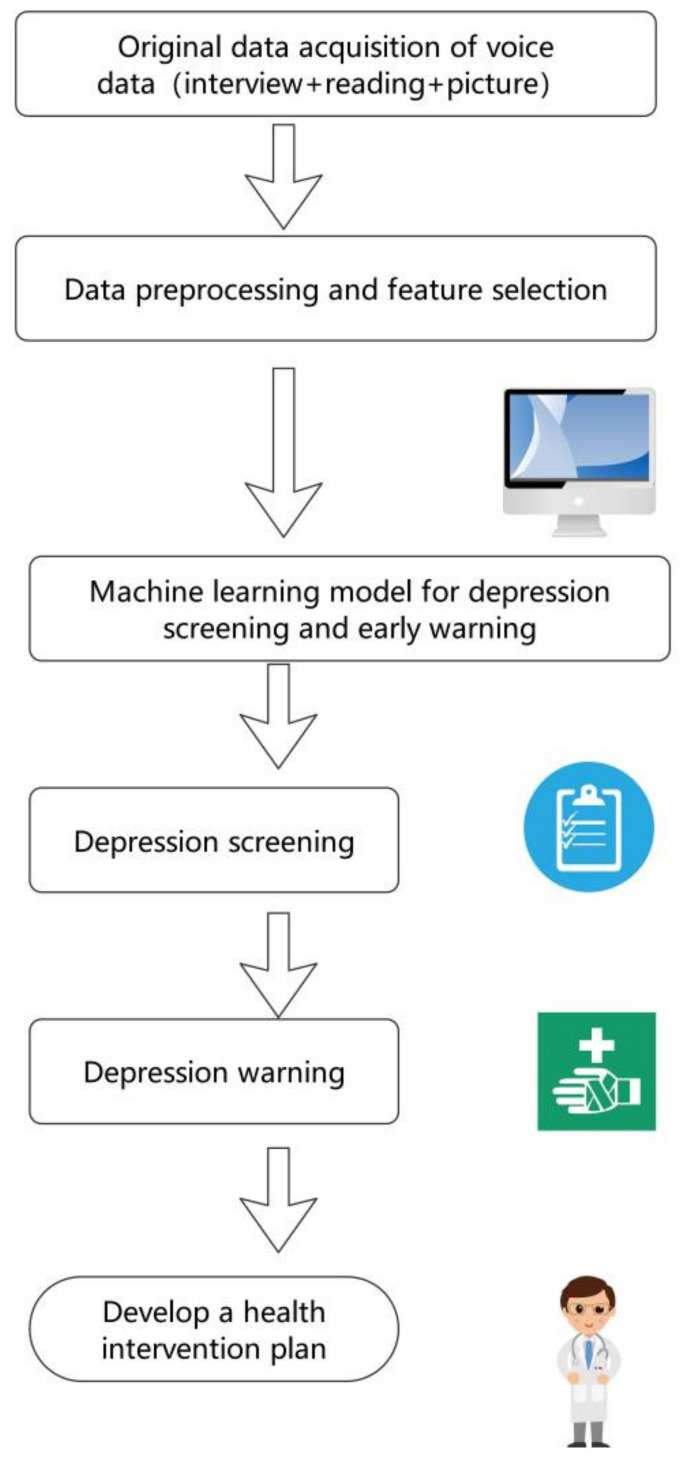
The depression screening and early warning model of this study.

The model of this study was shown in Figure 4. The voice data used for modeling adopted the authoritative public data set, which had passed the ethical review. Through data processing, a database containing 1479 voice feature samples was generated for modeling, and a recognition accuracy of 83.4% was achieved by using the CRT decision tree algorithm. According to the results of the screening, it was convenient and meaningful to give early warning to the patients who were identified as depressed, and ask professional doctors to make a health intervention plan for them. Therefore, the model can be used as a tool for public mental health management.

## 4. Discussion

This study discussed the method of using voice data for depression screening, and used the public data set of depression to build a decision tree model to verify the feasibility of the method. According to the results of screening, early warning and intervention can be carried out for individuals with depression in time to avoid further aggravation of depression, so as to prevent tragedies such as suicide caused by depression. The method of using voice data to predict depression has the advantages of convenient data acquisition and low cost. Voice data can be easily obtained through the microphone of mobile phones, without the need for professional doctors to operate. It only takes a little time and almost no cost. So, voice data are an ideal data source for depression screening [29,30].

The collected voice data can be divided into three forms. The experimental results showed that the model recognition accuracy was higher with the data of interview-type. Therefore, the interview-type of voice data is a good choice for depression screening. At the same time, through feature extraction, preprocessing of voice data and the optimization of the decision tree algorithm, the accuracy of the screening model reached 83.4%, which was better than the recent related research [31]. The comparison of the results is shown in Table 4. The results of Study 1 were the best results using voice data in the study. The result of Study 2 was the best result using a binary tree fusion model, and the performance of the model was evaluated only by accuracy. The precision and F1 score were not provided in Study 2. 

For voice features, we used 88 voice features of eGeMAPS, which had been proved to be effective in the field of emotion recognition in previous studies. By using the principal component analysis (PCA) dimension reduction algorithm to process data, reduce data dimension, and establish a screening model, the recognition ability of the model was reduced. So, all the 88 dimensional voice features were adopted. Through Python programming, we can extract the 88 dimensional voice features of each voice file, and then through the file read–write operation, we can write all the data features in a CSV file for the construction of the database.

From the perspective of the machine learning algorithm, the decision tree algorithm used in this study is an efficient classification algorithm, which can visually display the classification process in the form of spanning tree, and is suitable for the case of too many independent variables. At the same time, in view of the small number of voice data, 10-fold cross validation was used in the modeling process to prevent the model from over-fitting, and increase the robustness of the model.

If screened by the model as a depressive patient, psychotherapy can be used first, because this method has no side effects. In addition to the traditional psychotherapy methods, such as cognitive behavioral therapy (CBT), interpersonal relationship therapy (IPT), and psychodynamic therapy, artificial intelligence technology has also been used in the psychotherapy of depression in recent years, such as VR psychological intervention and mobile phone app psychological intervention [32]. In the severe cases of depression, drug treatment can be added. Drug therapy includes western medicine and traditional Chinese medicine. The advantage of traditional Chinese medicine in the treatment of depression is the small number of adverse reactions. People who are afraid of adverse drug reactions, and are unwilling to be treated, can choose traditional Chinese medicine for treatment.

The diagnosis of depression is very complex, there is no objective biomarker at present. The diagnosis mainly depends on doctors’ experience through interviews and scales. The acquisition of voice data is almost cost-free and noninvasive. It is very convenient to collect, and it does not require going to the hospital. Due to the high popularity of smart phones, you can easily record your own voice by using the recorder function of a smart phone. Therefore, the depression screening model based on voice data designed in this paper has the advantage of convenient and low cost. The traditional methods of depression diagnosis, based on doctor interview and scale, are time-consuming, high costing and subjective. After inputting the voice data, the computer model can make a diagnosis immediately. The accuracy of screening also has advantages compared with recent studies. So, the machine learning method designed in this paper is objective, accurate and fast. The screening model is the application of artificial intelligence in the field of the computer-aided diagnosis of depression, which represents a certain progress due to the above reasons.

For depression research, the existing public data set for depression is not large. For example, the MODMA data set used in this paper is relatively small. It is difficult to collect a large amount of medical data, due to the privacy of patients and the problem of patient compliance. With the development of computer technology, machine learning based on a small sample of medical data has made great progress in recent years. At present, there are some studies on small-sample machine learning, especially in the field of medical care. For example, in the study by Jiang et al. [11], using 4930 speech data of 170 samples, the authors extracted 1582 dimensional features and used machine learning to recognize depression. According to the description of MODMA, the 1479 feature data used in this paper are different. Multiple sampling of patients is also feasible because the disease is represented by a characteristic range. For example, the values of systolic blood pressure between 90 and 140 are all normal. If necessary, the dimension of feature can be reduced by PCA and other methods. The data involved in machine learning modeling are only depression tag information and voice feature information, and there is no speaker information. Therefore, the differentiation of depressive patients is based on the comprehensive expression of a series of digital voice features.

This study has the following limitations: First of all, the voice data used in the research are Chinese voice, which has limitations in the scope of application. Secondly, because medical data involves patient privacy, it is difficult to obtain data and the number of data are relatively small. This study also faces this problem. With the increase in model learning samples, the robustness of the model will be enhanced.

## 5. Conclusions

In this study, the machine learning method was used to establish a decision tree screening model for high-dimensional voice data, under different experimental conditions. According to the results of the model, early warnings can be given to the individuals with depression. The accuracy of the model recognition reached 83.4%. It showed that voice data could be used for rapid recognition and diagnosis of clinical depression. This method has the advantages of simple operation, low cost and fast data acquisition, which can be used for the management of public mental health. At the same time, according to the screening results, the patient can be carried out of the health management process of depression, including health data collection, health assessment, early warning and intervention, until the patient recovers.

## Figures and Tables

**Table 1:** Voice sample numbering.

Voice Types	Voice Sample Number
Interview	1–18
Passage reading	19
Vocabulary reading	20–25
Picture description	26–28
Thematic Apperception Test (TAT)	29

**Table 2:** Confusion matrix indicator formula.

Indicators	Formula
Accuracy	Accuracy = $\frac{TP+TN}{TP+TN+FP+FN}$
Precision	Precision = $\frac{TP}{TP+FP}$
Sensitivity (Recall)	Sensitivity = Recall = $\frac{TP}{TP+FN}$
Specificity	Specificity = $\frac{TN}{TN+FP}$
F1 Score	F1 Score = $\frac{2\times Precision\times Recall}{Precision+Recall}$

**Table 3:** Performance indicators of different database decision tree models.

	Accuracy	Precision	Recall	Specificity	F1 Score
interview	83.4%	81.9%	79.0%	86.8%	80.5%
reading	76.8%	80.9%	60.4%	89.2%	69.1%
picture	75.0%	66.7%	84.1%	68.1%	74.4%
all	83.4%	83.5%	76.8%	88.5%	80.0%

**Table 4:** Comparison of the results of similar studies.

	Accuracy	Precision	F1 Score
Our study (interview)	83.4%	81.9%	80.5%
Study 1 [31]	71%	77%	80%
Study 2 [12]	75.8%	Not provided	Not provided

## Data Availability

Publicly available datasets were analyzed in this study. This data can be found here: http://modma.lzu.edu.cn/data/index/ (accessed on 10 April 2021).
